# Effect of aspirin in patients with established asymptomatic carotid atherosclerosis: A systematic review and meta-analysis

**DOI:** 10.3389/fphar.2022.1041400

**Published:** 2022-12-07

**Authors:** Xianjin Hu, Yao Hu, Xiankun Sun, Ying Li, Ye Zhu

**Affiliations:** ^1^ Department of Cardiology, West China Hospital, Sichuan University, Chengdu, China; ^2^ Department of Traditional Chinese Medicine, Xiang He Community Healthcare Center, Chengdu, Sichuan, China; ^3^ Department of Nephrology, West China Hospital, Sichuan University, Chengdu, China; ^4^ Department of Cardiology, West China Fourth Hospital, Sichuan University, Chengdu, China

**Keywords:** aspirin, asymptomatic carotid atherosclerosis, carotid intima-media thickness, gastrointestinal bleeding, meta-analysis

## Abstract

**Background:** Aspirin is widely used as an antiplatelet agent for secondary prevention in patients with atherosclerotic cardiovascular disease. However, it remains unclear whether aspirin can prevent the progression of carotid atherosclerosis or reduce vascular events and all-cause death.

**Methods:** We performed a meta-analysis of the effect of aspirin in asymptomatic carotid atherosclerotic patients. Electronic databases including Pubmed, EMBase, ISI Web, Medline, Cochrane, and clinicaltrial.gov were searched for relevant randomized controlled trials. A total of five studies (841 individuals, 2,145 person-years) were included in this study. Two reviewers independently performed the study assessment and data extraction. Forest plots were used to assess the efficacy of aspirin. Egger’s test was used to evaluate publication bias.

**Results:** Aspirin did not alleviate the progression of carotid intima-media thickness (cIMT) compared with control patients (WMD: −0.05 mm, 95% confidence interval 95%CI: −0.12, 0.03). In subset analysis, aspirin was only associated with regression of cIMT when compared with the empty/placebo group (WMD: −0.10 mm, 95%CI: −0.18, −0.02). In type 2 diabetes mellitus, there were no statistical significance between groups (WMD: 0.10 mm, 95%CI: −0.31, 0.50). For the main vascular events and all-cause death, there were no differences between the aspirin group (RR: 0.73, 95%CI: 0.41, 1.31) and the control group (RR: 0.88, 95%CI: 0.41, 1.90). For outcome events, similar results were observed when patients were classified by different cIMT value (*p* > 0.05). The risk of gastrointestinal bleeding was similar between participants receiving and not receiving aspirin therapy (RR: 1.04, 95%CI: 0.07, 16.46).

**Conclusion:** In patients with asymptomatic carotid atherosclerosis, low-dose aspirin may slightly alleviate the progression of cIMT, but does not reduce vascular events and all-cause death.

**Systematic Review Registration:** https://www.crd.york.ac.uk/PROSPERO/, identifier PROSPERO

## Introduction

Atherosclerosis is a vital part of the chronic inflammatory reaction in the body, which involves a secondary autoimmune component (Egger et al., 2001; Kobiyama and Ley, 2018; Xu et al., 2018) that deteriorates over time. The normal artery wall has a triple-layer structure, with atherosclerotic plaques forming in the intima layer. Pathologically, atherosclerotic plaques show deposition of low-density lipoprotein particles, oxidative modification, monocyte migration, foam cell formation, smooth muscle migration, extracellular matrix molecule production, and necrotic core formation (Libby et al., 2019).

Previous studies have reported that carotid atherosclerosis is a surrogate biomarker for predicting further coronary heart disease risk (Geroulakos et al., 1994; Polak et al., 2011; Baldassarre et al., 2012; Bos et al., 2021). The global prevalence of elevated carotid intima-media thickness (cIMT), carotid plaques, and carotid stenosis in people aged 30–79 years in 2020 was 27.62%, 21.13%, and 1.5%, respectively (equivalent to 1066.70 million, 815.75 million, and 57.79 million people, respectively) (Song et al., 2020). Lipid-lowering agents and antiplatelet agents remain an integral part of atherosclerosis treatment. Numerous studies have demonstrated the efficacy of lipid-lowering agents (e.g., statins, Ezetimibe, and PSCK9 inhibitors), antiplatelet agents, and blood pressure and diabetes mellitus control in secondary prevention of atherosclerotic cardiovascular disease (Chapman, 2007; Baigent et al., 2009; Hackam, 2021). As for antiplatelet agents, it is important to assess the bleeding risk before using these treatments.

In 1828, willow was refined into yellow crystals and labeled salicin by a professor of pharmacy—this was the first report of identification and synthesis of the active ingredient of these ubiquitous trees (Fuster and Sweeny, 2011). Aspirin, a type of salicylic acid, is one of the most common antiplatelet pharmaceuticals in current clinical practice. Aspirin covalently and irreversibly inhibits cyclooxygenase and platelet thromboxane A2 biosynthesis (Maree and Fitzgerald, 2004; Kalra et al., 2013). Aspirin is widely used in the secondary prevention of coronary heart disease. In patients with transient ischemic attack or ischemic stroke, the employment of aspirin substantially reduces the risk of early recurrent stroke (Rothwell et al., 2016). Nevertheless, there was no apparent benefit for patients without prior ischemic events (Bavry et al., 2017). Furthermore, the benefit of aspirin in asymptomatic carotid atherosclerosis remains controversial (Paciaroni and Bogousslavsky, 2015). Bleeding events, including fatal or non-fatal gastrointestinal bleeding and intracranial hemorrhage, are a side effect of long-term aspirin use. Thus, considering the balance between antiplatelet and bleeding risk is important when administering this medicine to certain populations.

The aim of the present meta-analysis was to determine whether aspirin is beneficial in patients with asymptomatic carotid atherosclerosis, we assessed the efficacy of aspirin in cIMT regression, prevention of vascular events, and all-cause death, as well as the associated bleeding risk.

## Methods

This meta-analysis was initiated on 12 May 2022. After searching for studies in electronic databases, five randomized controlled trials involving 841 patients (mean duration: 2.55 years; 2,145 person-years) were included. Registration of the study protocol was done in advance in PROSPERO (No. CRD42022331783). PRISMA 2020 checklist was completed (Supplementary Material S1).

### Criteria for considering studies

Randomized controlled trials were eligible if they involved a comparison of an aspirin group *versus* an empty/placebo/other antiplatelet group in patients with asymptomatic carotid atherosclerotic diseases. We also examined reviews and meta-analyses of antiplatelet agents and carotid atherosclerosis as additional resources. Exclusion criteria included 1) Laboratory studies, cohort studies, cross-sectional studies, case-control studies, case reports, letters, commentaries, and summaries, 2) Studies performed on patients with acute myocardial infarction, coronary artery disease, angina pectoris, stroke, lower extremities atherosclerotic occlusive disease, or other complications that involved antiplatelet therapy, 3) Studies that did not report the change in cIMT, carotid plaques, vascular events, deaths, or hemorrhagic events, and 4) Studies where data could not be extracted.

### Search methods for identification of studies

Electronic databases including Pubmed, EMBase, ISI Web, Medline, Cochrane, and clinicaltrial.gov were searched by using the Mesh or Title/Abstract of (“Aspirin” OR “Antiplatelet”) AND (“CIMT” OR “Carotid Intima-Media Thickness” OR “Carotid plaques” OR “Carotid atherosclerosis”) from inception to May 2022. Reviews, meta-analyses, and the references of the identified studies were examined to search for additional resources. There were no restrictions in languages and regions.

### Main outcomes

Primary outcomes in this meta-analysis were 1) The change in cIMT or carotid plaques, 2) Cardiovascular events (e.g., acute myocardial infarction, unstable angina, and progression of coronary artery disease), 3) Cerebrovascular events (e.g., transient ischemic attacks, ischemic stroke, and hemorrhagic stroke), and 4) All-cause death. The secondary outcomes were gastrointestinal bleeding events.

### Data collection and synthesis

**FIGURE 1:**
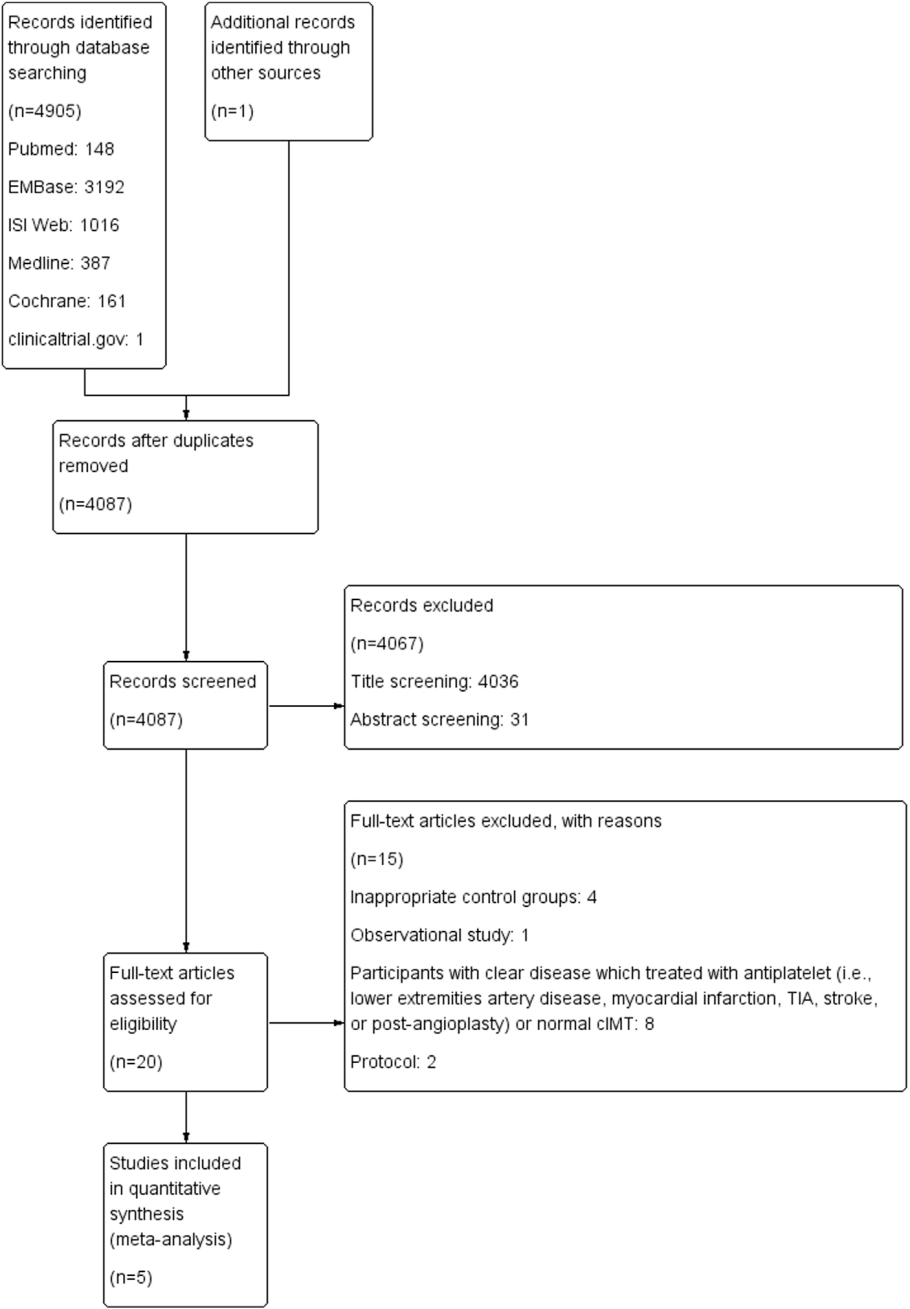
Flow diagram.

All studies collected through electronic databases were imported into EndNote X9 and duplicate records were removed. Two reviewers (Xianjin Hu and Yao Hu) separately examined the title, abstract, and entire text of each study. Discrepancies were resolved by consensus or a third author adjudication (Xiankun Sun). These procedures are shown in Figure 1.

Based on a predetermined form, data were separately retrieved by two reviewers. Conflicts were settled by consensus or adjudication by a third author. The following data were collected from each study: first author, publication date, participants’ characteristics (male proportion, age), country of study, sample sizes of the various groups, interventions and duration in each group, and the main outcomes (cIMT, cardio-cerebrovascular events, deaths, and hemorrhagic events).

We determined the change in the mean cIMT between baseline and the study end by multiplying the mean cIMT value (per year change) by the length of the follow-up period, which was reported in one study (Kodama et al., 2000). A similar calculation was used to compute the standard deviation (SD). The Excel spreadsheet provided by the Cochrane website was used to calculate the SD using the *p*-value or standard error, allowing us to extract SD from other studies that did not disclose the SD of changes in cIMT values.

### Quality assessment

Two reviewers independently evaluated the risk of bias in the included trials using the Cochrane Collaboration tool (random sequence generation, allocation concealment, blinding of participants and personnel, blinding of outcome assessment, incomplete outcome data, selective reporting, and other bias). Conflicts were settled by consensus or adjudication by a third author.

### Statistical analysis

All analyses were performed with Review Manager (v5.4.1; RevMan, the Cochrane collaboration, Oxford, United Kingdom) and Stata 17. For continuous variables, data are presented as weighted mean difference (WMD) with 95% confidence intervals (95%CI). For binary variables, data are presented as relative risk (RR) and 95%CI. A random effects model was used for statistical analysis because of the clinical and methodological variability across the trials. Heterogeneity across trials was assessed using the standard Chi-square test (significance set at *p* < 0.05) and the I^2^ statistic (significance set at I^2^ > 50%) Egger’s test was performed to assess the potential for publication bias.

## Results

### Features of the selected studies

Our meta-analysis included five randomized controlled trials with 841 patients. A flow diagram of the selection procedure is shown in Figure 1. After the initial search, a total of 4,905 articles were included (148 from Pubmed, 3,192 from EMBase, 1016 from ISI Web, 387 from Medline, 161 from Cochrane, and one from clinicaltrial.gov). Only one eligible study was obtained from the reference lists. After eliminating duplicates and screening the title and abstract, full text assessments were performed in 20 trials. Of these, five studies were included in the final quantitative analysis.

Detailed information from the five studies is shown in Table 1. All studies were designed as a randomized conthrolled trial. Four of the studies used a 1:1 allocation for the aspirin group and control group, while one study used a 1:2 allocation. Two studies enrolled participants with type 2 diabetes mellitus, and one study enrolled patients with hypertension. There were two studies eliminating the patients treated with anticoagulation. The rest of trials did not mention the information of using anticoagulation or not. All interventional groups received aspirin therapy, although the doses were different (75–325 mg daily). Two studies used an empty group for comparisons (one with cilostazol and one with placebo), and one study used a standard management group (including diet and exercise). Four of the studies were followed up for 2.3–3 years, while one study was followed up for 6 months. All included patients had asymptomatic increased cIMT, carotid stenosis, or carotid plaques.

**TABLE 1:** Features of selected studies.

Author (Year)	Country	Sample size (Asp/Con)	Demography (Male%/Age)	Complication	Anticoagulation	Intervention/Control	Duration	Carotid atherosclerosis	Main outcomes
Côté et al. (1995)	US	188/184	44.7%/65.9 ± 8.5	NR	Exclusion	Asp 325 mg vs. Placebo	2.3 years	Asymptomatic carotid stenosis >50%	Vascular events, death
Kodama et al. (2000)	Japan	40/74	57.5%/65.5 ± 1.0	T2DM	NR	Asp 81 mg vs. Empty	3 years	cIMT >1.1 mm	CIMT
Peng et al. (2006)	China	82/80	58.53%/63 ± 13	HP	NR	Asp 75 mg + Sim 20 mg vs. Empty	3 years	cIMT ≥ 1.0 mm	CIMT, lipids, plaque score, vascular events
Belcaro et al. (2020)	Italy	86/60	60.47%/63.2 ± 2.5	NR	Exclusion	Asp 100 mg + SM vs. SM	3 years	cIMT ≥ 1.5 mm or Asymptomatic carotid stenosis >50%	CIMT, cardiovascular events
Lee et al. (2020)	Korean	23/24	69.57%/59.1 ± 8.8	T2DM	NR	Asp 100 mg vs. Cilostazol 200 mg	6 months	Carotid plaques	CIMT, plaque volume

Asp, aspirin; Con, control group; Sim, simvastatin; SM, standard management; NR, not reported; T2DM, type 2 diabetes mellitus; HP, hypertension; cIMT, carotid intima-media thickness.

### Main outcomes

**FIGURE 2:**
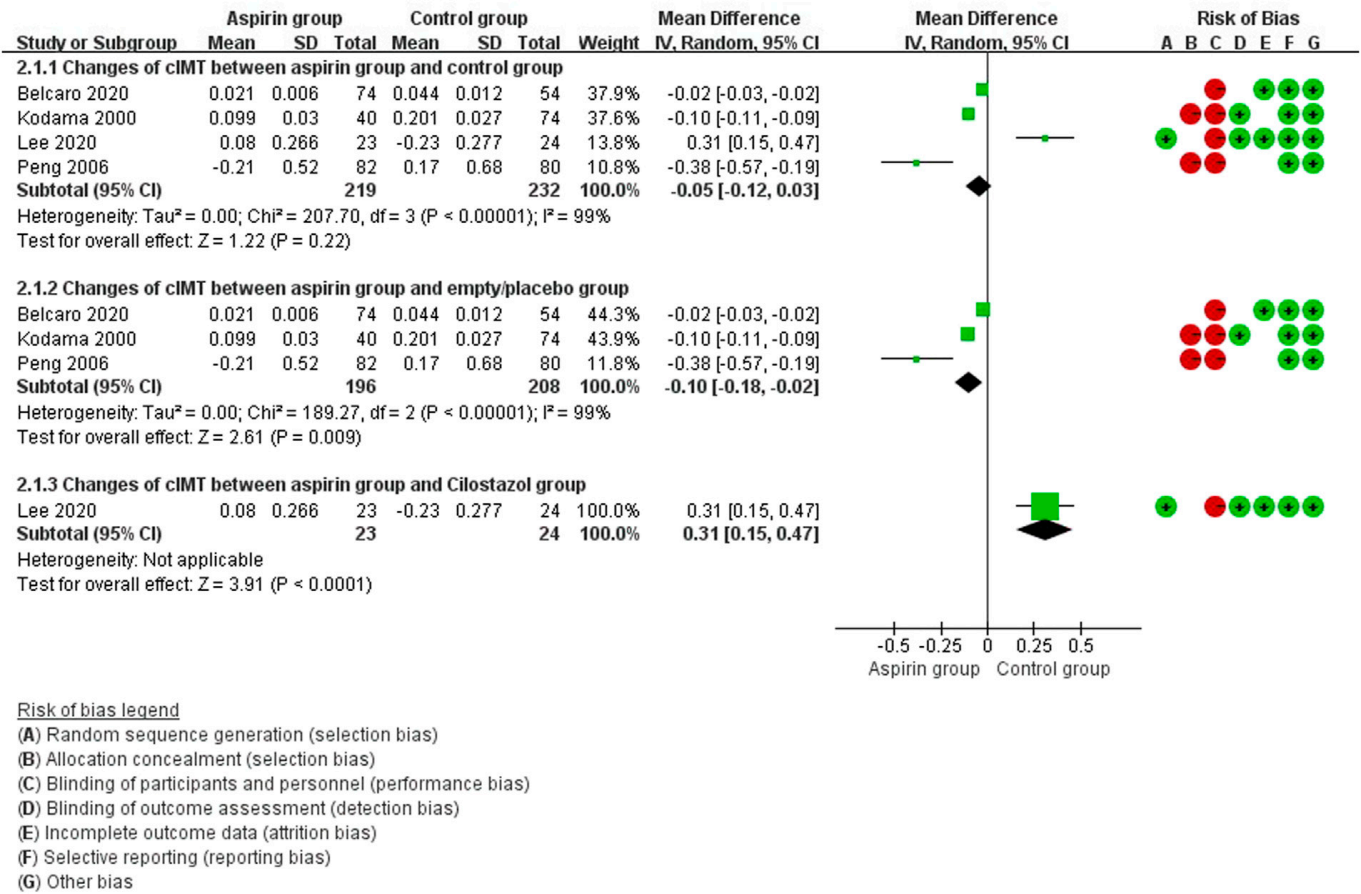
Forest plot of changes in carotid intima-media thickness (cIMT) between the aspirin group and the control group.

In pooled analysis, aspirin therapy was not associated with changes in cIMT values compared with the control group (WMD: −0.05 mm, 95%CI: −0.12, 0.03). In subset analysis, aspirin alleviated the progression of cIMT when compared with the empty/placebo group (WMD: −0.10 mm, 95%CI: −0.18, −0.02), while the efficacy of cilostazol might be better that of aspirin (WMD: 0.31 mm, 95%CI: 0.15, 0.47) (Figure 2).

**FIGURE 3:**
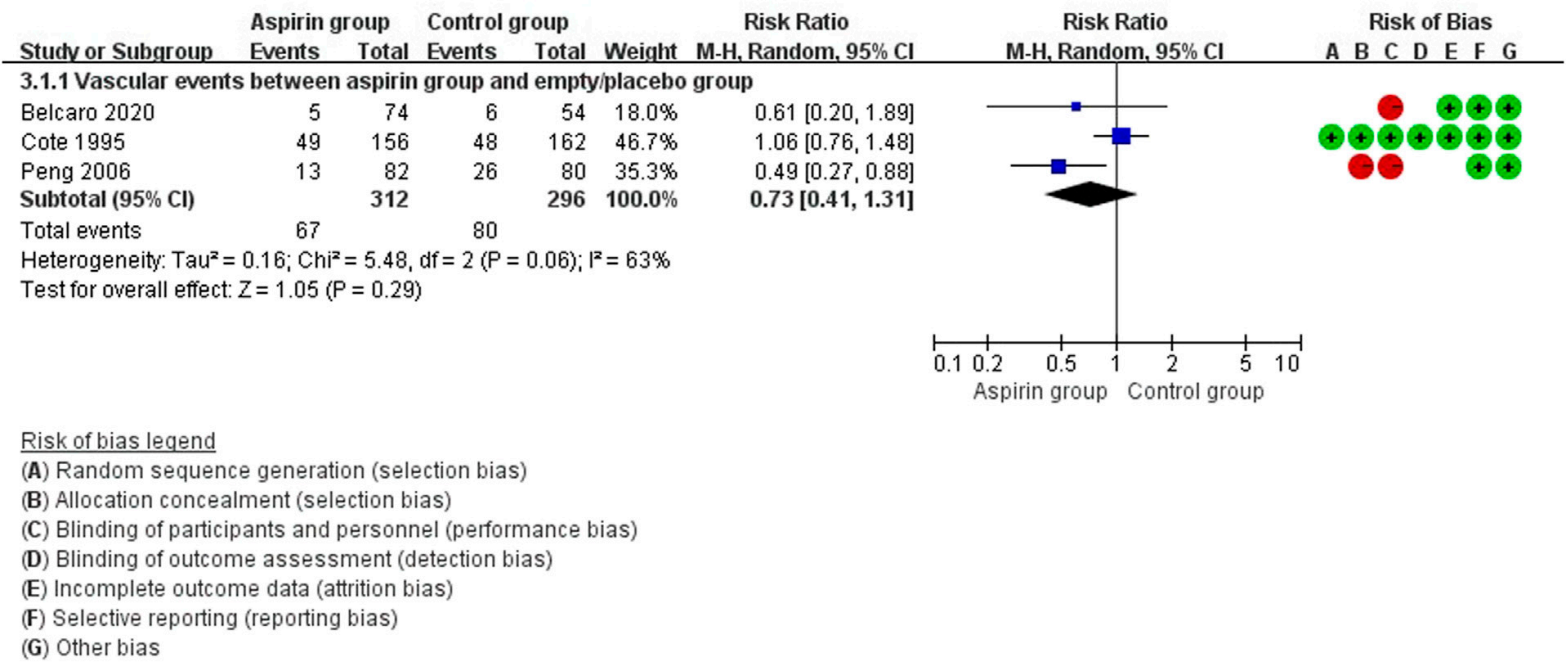
Forest plot of vascular events between the aspirin group and the control group.

**FIGURE 4:**
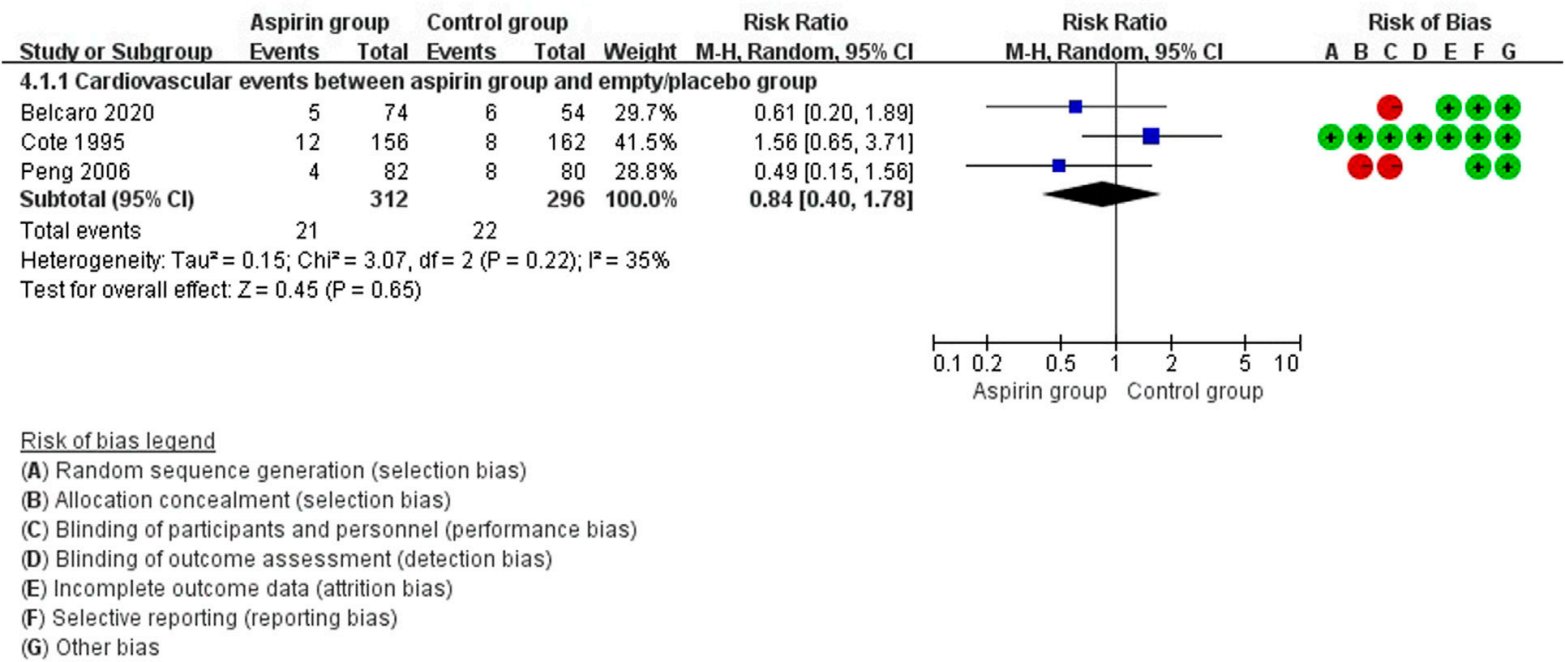
Forest plot of cardiovascular events between the aspirin group and the control group.

**FIGURE 5:**
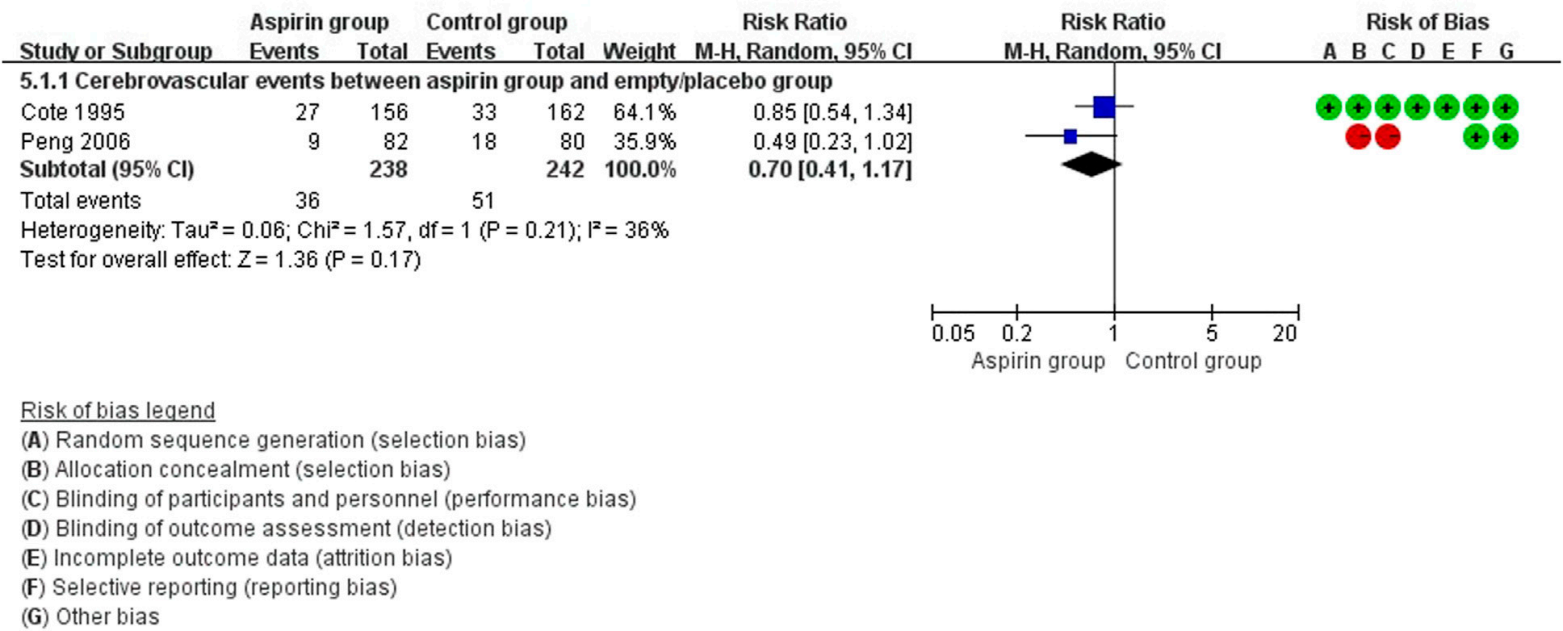
Forest plot of cerebrovascular events between the aspirin group and the control group.

**FIGURE 6:**
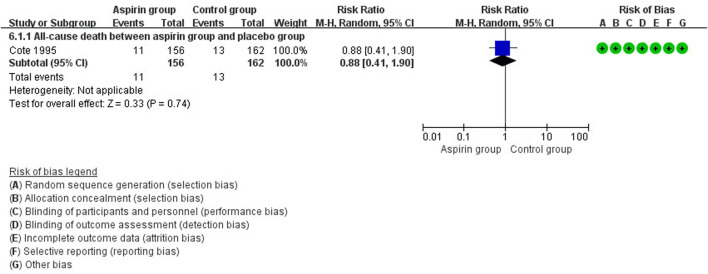
Forest plot of all-cause death between the aspirin group and the control group.

**FIGURE 7:**
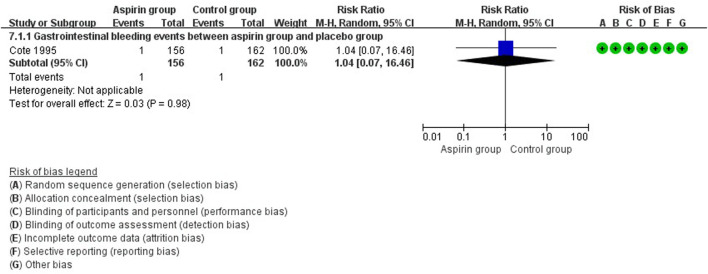
Forest plot of gastrointestinal bleeding events between the aspirin group and the control group.

There were no differences in vascular events between the aspirin group and the control group (RR: 0.73, 95%CI: 0.41, 1.31) (Figure 3). Separate analysis of cardiovascular events (Figure 4) and cerebrovascular events (Figure 5) also showed no difference between the aspirin and control groups. Aspirin did not reduce all-cause death (RR: 0.88, 95%CI: 0.41, 1.90) (Figure 6) in one study that reported mortality. Finally, there were no differences in gastrointestinal bleeding events between patients receiving or not receiving aspirin therapy (RR: 1.04, 95%CI: 0.07, 16.46) (Figure 7).

**FIGURE 8:**
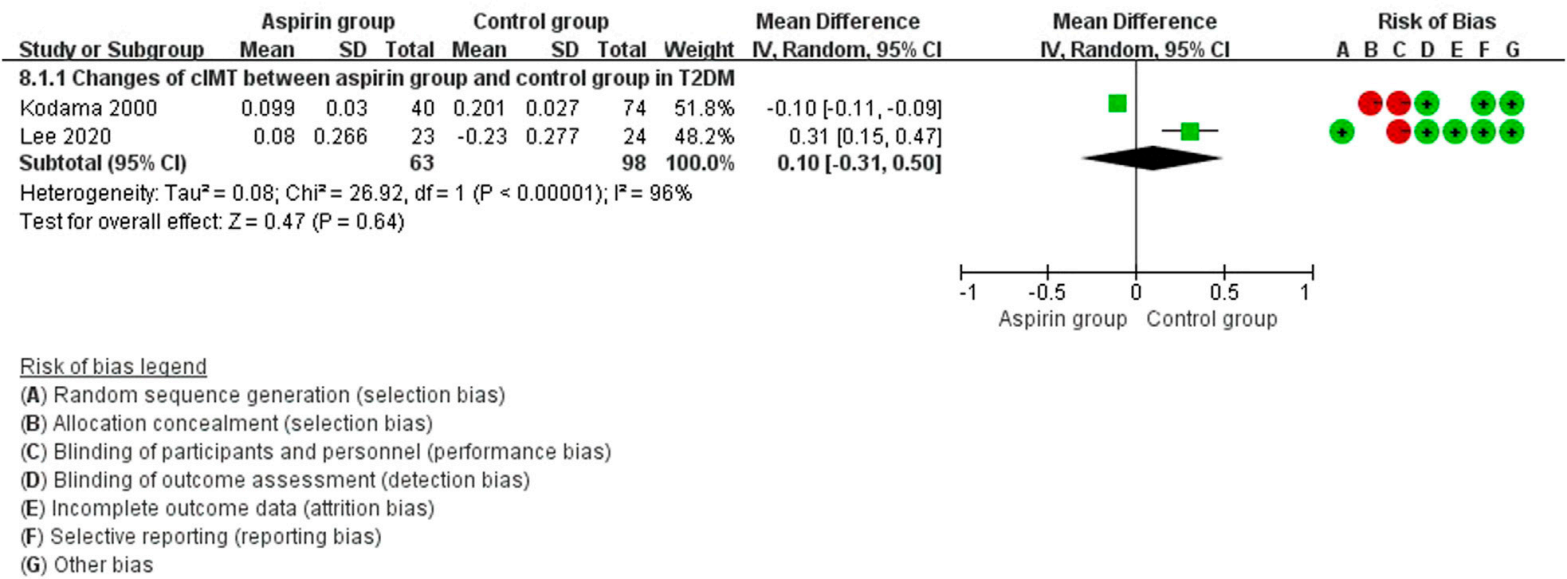
Forest plot of changes in carotid intima-media thickness (cIMT) between the aspirin group and the control group in type 2 diabetes mellitus.

**FIGURE 9:**
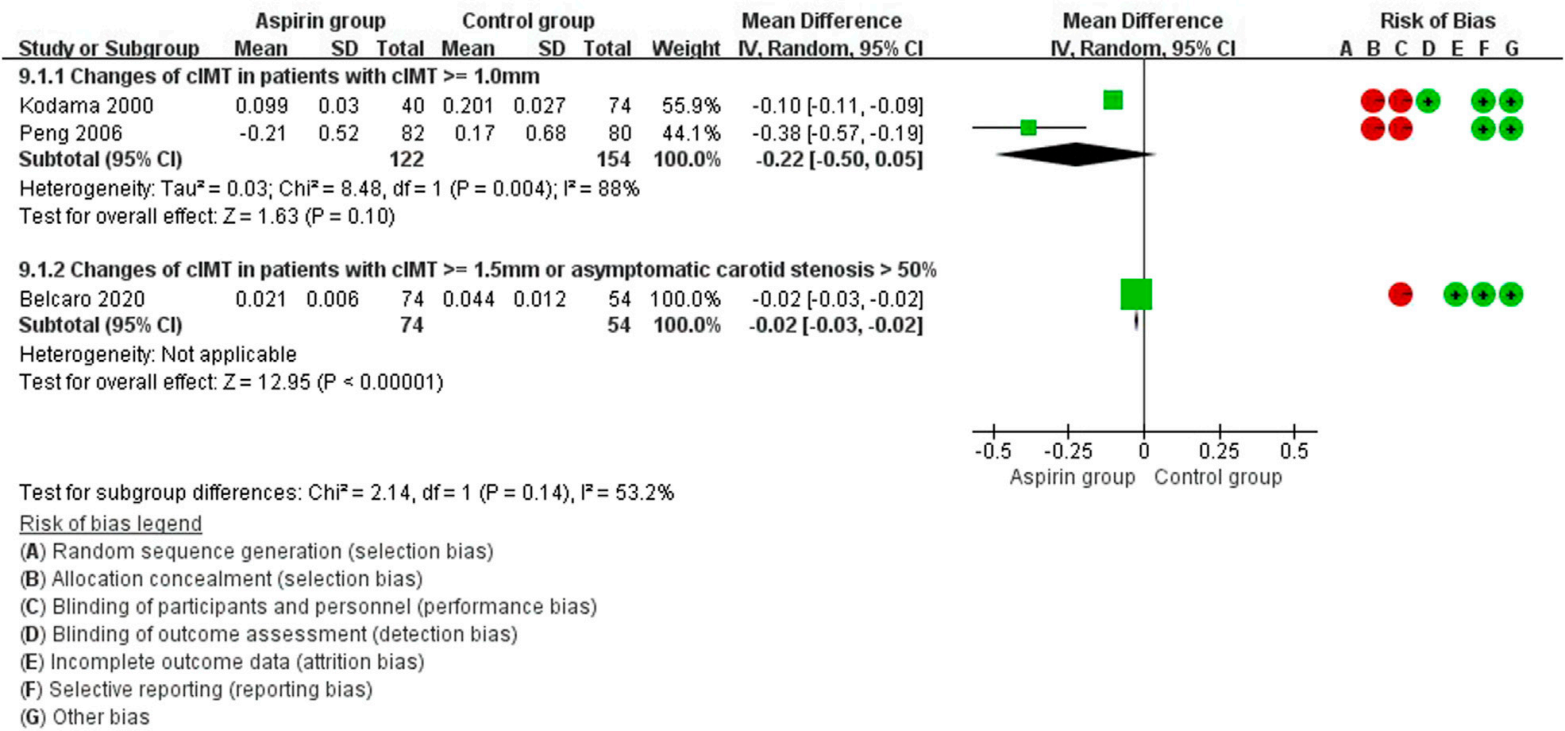
Forest plot of changes in carotid intima-media thickness (cIMT) in patients with cIMT ≥ 1.0 mm.

**FIGURE 10:**
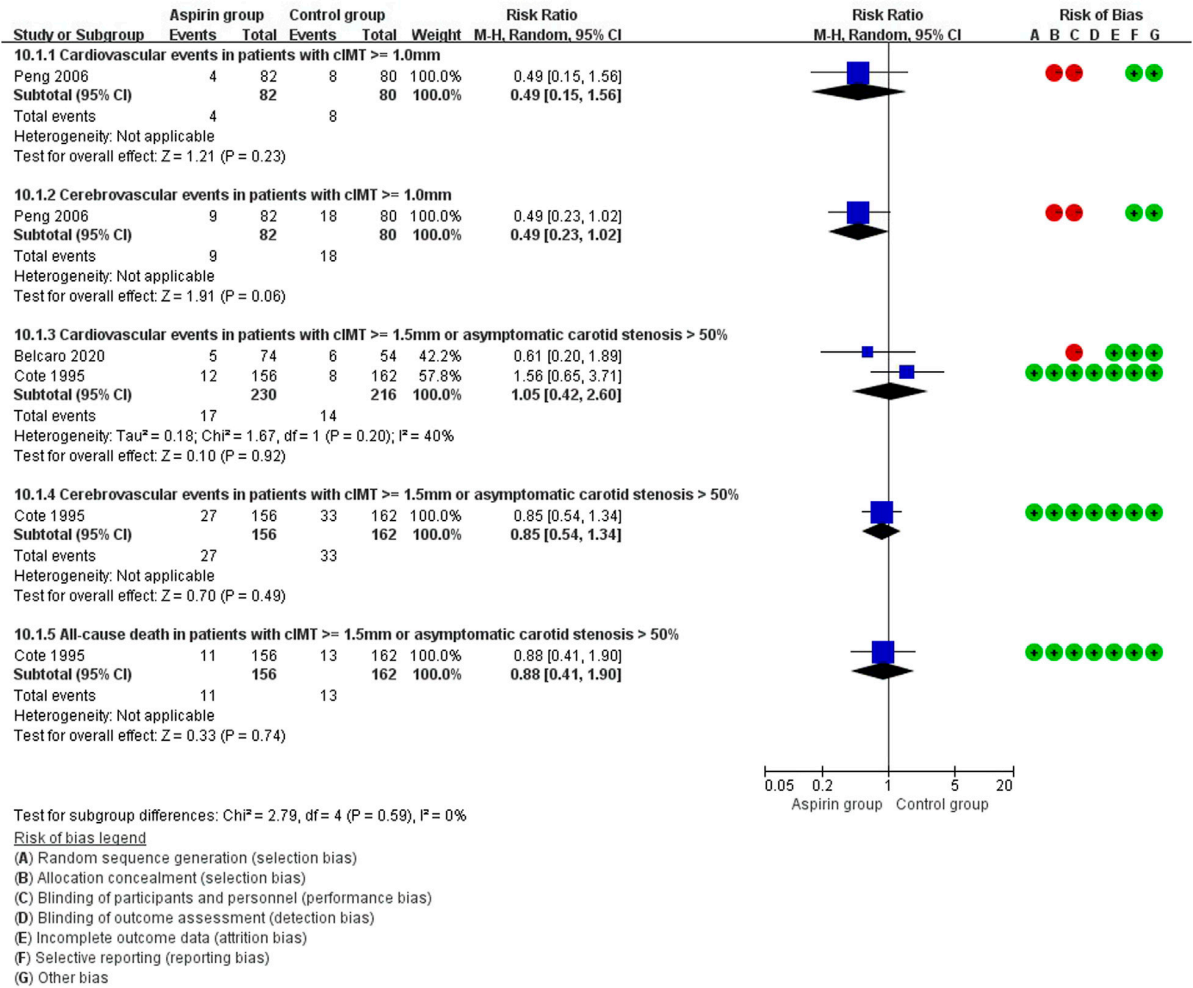
Forest plot of outcome events in patients with different carotid intima-media thickness (cIMT) or carotid stenosis.

In the subset analysis of type 2 diabetes mellitus, there were no statistical significance between groups (WMD: 0.10 mm, 95%CI: −0.31, 0.50) (Figure 8). In patients with cIMT ≥ 1.0 mm, cIMT reduced slightly but no statistical significance (WMD: −0.22 mm, 95%CI: −0.50, 0.05) (Figure 9). Similar results were observed in patients with cIMT ≥ 1.5 mm or asymptomatic carotid stenosis >50% (WMD: −0.02 mm, 95%CI: −0.03, −0.02) (Figure 9). For the outcome events, including cardiovascular events, cerebrovascular events and all-cause death, there were no statistical significance between aspirin group and control group when patients classified by different cIMT (Figure 10).

### Publication bias

**FIGURE 11:**
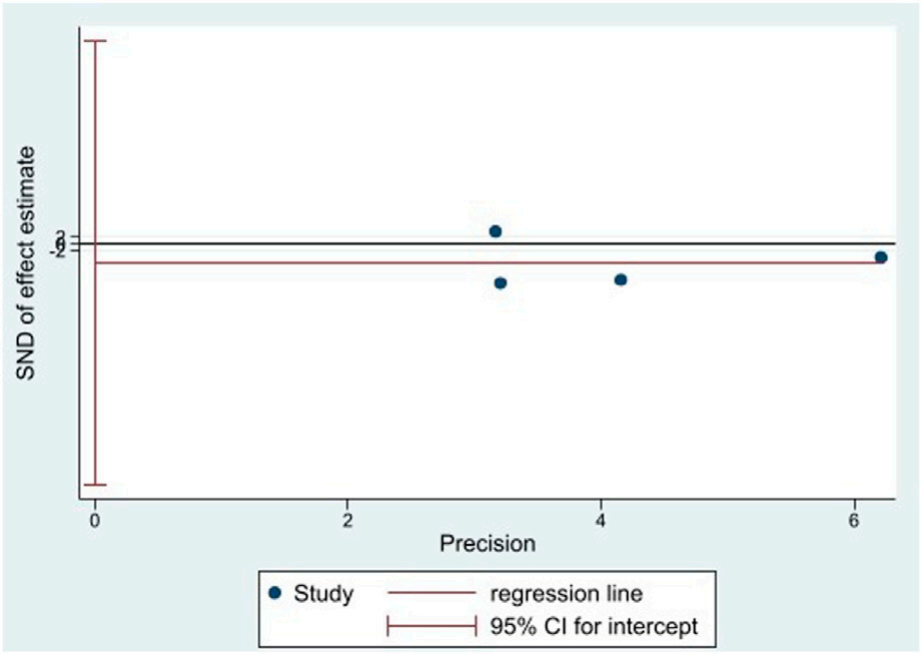
Egger’s test for carotid intima-media thickness (cIMT).

**FIGURE 12:**
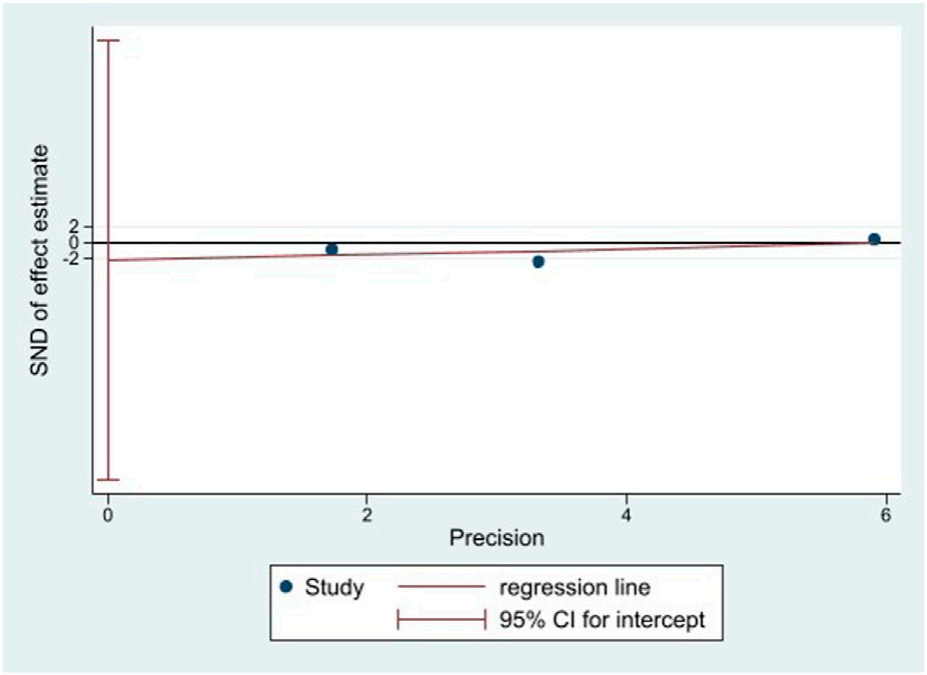
Egger’s test for vascular events.

Because of the small number of trials involved in this meta-analysis, it was difficult to assess publication bias using a funnel plot. Thus, we utilized the Egger’s test (Figures 11, 12). There was no significant publication bias in the continuous (*p* = 0.744) and binary (*p* = 0.483) variables.

## Discussion

The aim of this meta-analysis was to determine the efficacy of antiplatelet agents in patients with asymptomatic carotid atherosclerosis. Our main finding was that aspirin did not reduce the incidence of vascular events and all-cause death in patients with asymptomatic carotid atherosclerosis. Nevertheless, compared with an empty/placebo group, aspirin marginally reduced the progression of carotid atherosclerosis. Furthermore, during the follow-up period, there was no effect of aspirin on gastrointestinal bleeding risk.

The therapeutic strategies used in the included studies were based on the presence of symptoms caused by cerebral circulation insufficiency. According to the European Stroke Organization’s guidelines (Bonati et al., 2021), carotid endarterectomy is recommended for patients with moderate–severe symptomatic carotid artery stenosis (50%–99%). By contrast, patients with mild symptomatic stenosis (<50%) are not advised to accept endarterectomy. The European Society of Cardiology guidelines for diagnosis and treatment of peripheral arterial disease (Aboyans et al., 2018) suggest that medical therapy is better than surgery in patients with asymptomatic carotid stenosis (<60%), symptomatic carotid stenosis (<50%), and near occlusion or occlusion. Indeed, the 1-year risk of stroke or death was often lower with intensive medical therapy (approximately 0.5%) than with either carotid endarterectomy or stenting in asymptomatic carotid stenosis patients (Spence, 2020). Thus, most of patients with asymptomatic carotid stenosis should accept medical therapy to reduce vascular events morbidity and mortality. According to our meta-analysis, however, aspirin might not be necessary for these patients when no complication proposed to accept antiplatelet agents.

According to the pathological process of atherosclerosis, lipid deposition, oxidation, and platelet accumulation are critical events during plaque formation and the onset of complications (Reininger et al., 2010). These are also therapeutic targets in clinical practice. For example, aspirin acts as an antiplatelet agent by reducing thromboxane A_2_ synthesis, and has anti-inflammatory properties involving inhibition of cyclooxygenase activity (Vane and Botting, 2003; Yasuda et al., 2008). In a recent network meta-analysis evaluating the efficacies of several medications on cIMT progression (Huang et al., 2019), phosphodiesterase III inhibitors were the most efficient in reducing the annual mean cIMT, followed by calcium channel blockers, platelet ADP inhibitors, and cyclooxygenase inhibitors (WMD: −0.033 mm per year). However, the network analysis only included two studies of aspirin. In the present meta-analysis, we found that aspirin may slightly reduce the progression of carotid atherosclerosis. The efficacy of aspirin on cIMT may relate to its anti-inflammatory properties (Feldman et al., 2001; Arazi and Badimon, 2012). Other common antiplatelet agents (e.g., cilostazol and clopidogrel) were also reported to be beneficial in preventing carotid atherosclerosis progression (Geng et al., 2012; Takeda et al., 2012). Indeed, the development of symptoms caused by carotid atherosclerosis differed from the progression of carotid atherosclerosis (Park et al., 2016). Nevertheless, previous meta-analyses did not report whether small changes in cIMT were associated with reversal of endpoint events.

The key outcome assessed in the present study was reversal of the endpoints. However, aspirin had no effect on the incidence of vascular events and all-cause death in asymptomatic carotid atherosclerosis patients. Furthermore, the endpoints were not reversed regardless of whether the cIMT value was higher than 1.0 mm or 1.5 mm. Similar outcomes were reported in randomized controlled trials. For example, in a large primary-prevention trial of cardiovascular disease and cancer in women, aspirin reduced the risk of stroke, but had no effect on risk of myocardial infarction or death from cardiovascular causes (Ridker et al., 2005). Comparable results were also reported in patients with type 2 diabetes mellitus (Ogawa et al., 2008), older adults (McNeil et al., 2018), and in Japanese patients >60 years old with atherosclerotic risk factors (Ikeda et al., 2014). Furthermore, in asymptomatic atherosclerotic patients with a low ankle brachial index, aspirin administration had no effect on vascular events compared with placebo (Fowkes et al., 2010). Thus, there is no strong evidence for routine aspirin administration in asymptomatic carotid atherosclerosis patients. Long-term aspirin therapy is also associated with risk of bleeding (Zheng and Roddick, 2019; Gresele et al., 2020). For example, in a recent randomized controlled trial of patients with diabetes without evident cardiovascular disease, although aspirin prevented serious vascular events, this was largely counterbalanced by the bleeding hazard (Bowman et al., 2018). Use of other antithrombotic treatments (e.g., vitamin K antagonists and salicylates) was also suggested to cause intra-plaque hemorrhage that may induce vascular events (Mujaj et al., 2018). As such, it is necessary to identify vulnerable plaques and classify the risk of bleeding, especially in patients with a low-risk of cardiovascular disease (Faggiano et al., 2017; Gaziano et al., 2018). In the present study, there was no difference in the risk of gastrointestinal bleeding between patients receiving short-term aspirin and those not.

There are several limitations of our meta-analysis. The first was the lack of relevant randomized controlled trials, which reduces the accuracy and extrapolation of our findings. Second, because carotid atherosclerosis was detected *via* Doppler ultrasound in all included trials, it was difficult to identify and classify the vulnerable plaques. Thus, our conclusions may not be appropriate for patients with a high risk of future vascular events, even if they show no ischemia symptoms. Third, the I^2^ value was >50% in part of our study, suggestive of partial heterogeneity. Fourth, the aspirin doses ranged from 75 to 325 mg daily, which may change the antiplatelet properties and risk of bleeding. Finally, the short follow-up duration of the trials included in this study may underestimate the efficacy of aspirin in asymptomatic carotid atherosclerosis patients. Thus, large and long-term cohort studies and randomized controlled trials are required to confirm the effects of aspirin in this population.

## Conclusion

Low-dose aspirin was unable to reduce vascular events and all-cause death in patients with asymptomatic carotid atherosclerosis, with only a minor improvement in the progression of cIMT. Nevertheless, the risk of gastrointestinal bleeding was not increased after short-term aspirin treatment. However, the long-term efficacy of antiplatelet agents remains unclear in patients with asymptomatic carotid atherosclerosis. For patients with vulnerable plaques or a high risk of future cardiovascular events, treatment with antiplatelet agents can be considered for patients with a low risk of bleeding.

## Data Availability

The original contributions presented in the study are included in the article/Supplementary Material, further inquiries can be directed to the corresponding author.

## Abbreviations

CI, confidence intervals; CIMT, carotid intima-media thickness; RR, relative risk; SD, standard deviation; WMD, weighted mean difference.
